# Case Report: A case of pediatric reninoma and literatures review

**DOI:** 10.3389/fmed.2026.1741277

**Published:** 2026-03-16

**Authors:** Wanqing Zhao, Xiaohui Yuan, Lei Wang, Xue Yan, Hemeng Chong, Yuejing Li, Yanan Zhang

**Affiliations:** 1 Department of Pediatrics, The Second Hospital of Hebei Medical University, Shijiazhuang, China; 2 Department of Pediatrics, Shijiazhuang Maternal and Child Health Hospital, Shijiazhuang, China

**Keywords:** children, hypertension, hypokalemia, renin—angiotensin—aldosterone system (RAS), reninoma

## Abstract

**Objective:**

To investigate the clinical manifestations, pathological characteristics, diagnosis, treatment, and prognosis of reninoma.

**Methods:**

A retrospective analysis was conducted on the clinical data of one reninoma patient admitted in 2023, supplemented by an analysis of 62 reninoma cases diagnosed and treated in China between 2016 and May 2025, as reported in the literature.

**Results:**

The patient was a 10-year-old female admitted for hypertension detected during a physical examination. With normal serum potassium levels, bilateral renal vein sampling confirmed atypical reninoma. Under ultrasound guidance, radiofrequency ablation was performed on the right renal tumor, and pathological examination confirmed the diagnosis. Two years postoperatively, the patient presented again with elevated blood pressure and underwent partial right nephrectomy, after which blood pressure normalized. Reviewing the literature, among the 62 reported cases in China, 44 were typical reninoma, 16 were atypical reninoma (patients presented only with hypertension and normal serum potassium levels), and 2 were non-functional reninomas.

**Conclusion:**

Young women presenting with high renin and aldosterone, with or without hypokalemia, should raise suspicion for reninoma. Diagnosis requires integration of clinical presentation, imaging findings, and pathological characteristics. Curative treatment is achievable through tumor resection preserving renal units.

## 1 Introduction

Reninoma, also known as juxtaglomerular cell tumor, is a rare benign kidney tumor primarily occurring in young women. This tumor can cause excessive activation of the renin-angiotensin-aldosterone system by secreting renin, leading to significant secondary hyperaldosteronism. The aldosterone-induced sodium retention and potassium excretion result in renal potassium loss, causing hypokalemia. This combination produces the clinical symptoms of “high renin, high aldosterone, high blood pressure, and low serum potassium” (1). This paper reports a pediatric case of a reninoma diagnosed and treated at our institution. It also summarizes data from Chinese reninoma patients documented in the literature over the past decade, aiming to enhance clinicians’ awareness of this condition.

## 2 Medical history

A 10-year-old female patient was admitted to our institution in September 2023 presenting with “elevated blood pressure detected over 1 year ago and headache for 4 days.” During a physical examination 1 year before admission, blood pressure readings of 130–150/90–120 mmHg were noted, but no regular treatment was initiated. Four days prior to admission, she developed headaches without vomiting, seizures, palpitations, dyspnea, urinary urgency, frequency, or oliguria. Blood pressure monitoring revealed readings of 180–190/120–130 mmHg, prompting referral to our institution.

The patient was the second child of three, delivered full-term vaginally. She has no history of illness and normal growth and development. No family history of similar conditions exists.

Physical examination: T 36.5°C, P 96 bpm, R 24 bpm, Height 140 cm, Weight 32 kg. BP: left upper limb 150/101 mmHg, right upper limb 141/103 mmHg, left lower limb 149/80 mmHg, right lower limb 155/87 mmHg (1 mmHg = 0.133 kPa). She was conscious and alert, responsive. She had no jaundice, rash, or petechiae on skin. Pharynx was without hyperemia; tonsils were not enlarged; trachea was midline; thyroid was not enlarged. Clear breath sound in both lungs; there was no dry or wet rales heard. Heart rated 96 beats/min, and it had regular rhythm, she had strong heart and no murmurs heard. She had flat and soft abdomen, no tenderness, rebound tenderness, or muscle guarding. Her liver and spleen were not palpable. She had no tenderness in renal areas, no masses palpable. Her bowel sound present and normal. Her neurological examination revealed no significant abnormalities.

Supportive tests: Routine blood, urine, and stool tests; liver and kidney function; cardiac enzymes; erythrocyte sedimentation rate; autoantibodies—no abnormalities detected. Orthostatic aldosterone test indicated elevated renin, angiotensin, and aldosterone levels; specific results are shown in Table 1. Electrolytes showed sodium 139 mmol/L, potassium 4.77 mmol/L. No significant abnormalities were observed in adrenocorticotropic hormone (ACTH), cortisol, 17α-hydroxyprogesterone, or 24-h urinary vanillylmandelic acid. 24-h urinary free cortisol was < 10.00 μg/dL. A 24-h urine calcium-to-creatinine ratio: 0.4 mmol/mmol; creatinine clearance(Schwartz) = 109.61 mL/min/1.73 m^2^; 24-h urine potassium: 29.41 mmol; urinary potassium excretion rate: 13.37 nmol/L; 24-h urine potassium-to-creatinine ratio: 7.26 mmol/mmol.

**TABLE 1:** Three-point results for supine and standing hypertension.

Position	Renin (pg/mL) (2.4–32.8)	Angiotensin II (ng/mL/h) (0.41–2.44)	Aldosterone (pg/mL) (10–160)	Aldosterone/renin ratio (0–37)
Standing	182.09	18.51	396.69	2.20
Lying	174.2	17.7	346.87	1.99

Twenty four hour ambulatory blood pressure monitoring showed: systolic peak 218 mmHg, diastolic peak 126 mmHg, indicating: 1. Elevated mean blood pressure; 2. Severe loss of blood pressure load; 3. Absence of circadian rhythm. Cardiac ultrasound revealed: mild enlargement of the left atrium, mild aortic valve insufficiency, and mild mitral and tricuspid valve insufficiency.

**FIGURE 1:**
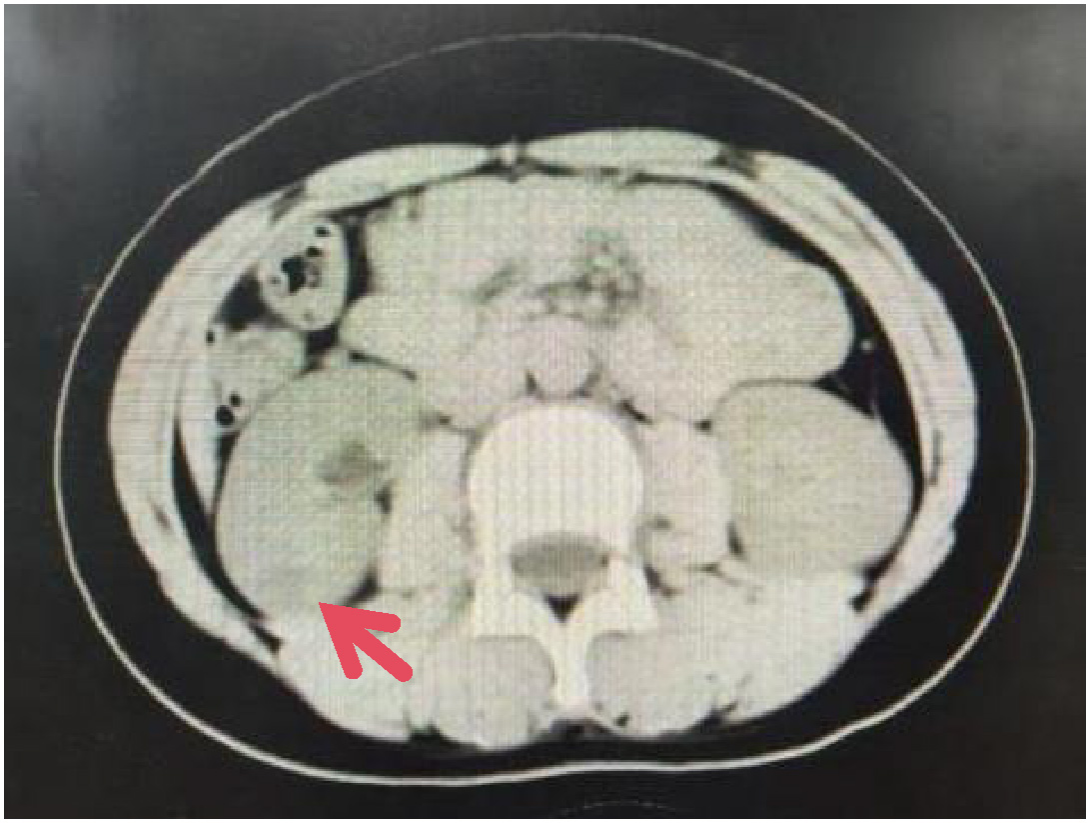
Kidney CT scan with contrast enhancement.

**FIGURE 2:**
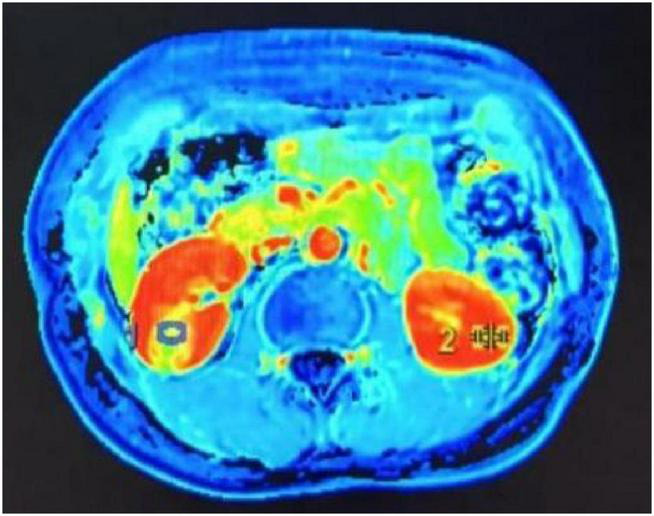
Renal dynamic scan + glomerular filtration rate.

Imaging examinations: CT of the abdominal aorta and kidneys with 3D reconstruction showed bilateral renal arteries with double-vessel imaging, with some lumens appearing slender. MR angiography of the renal arteries demonstrated bilateral adrenal arteries; the right bilateral renal arteries were slightly slenderer than the contralateral side. Cystic lesion in the lower pole of the right kidney. No significant abnormalities in bilateral renal artery blood flow. No structural or flow abnormalities detected in bilateral carotid arteries, upper limb arteries, lower limb arteries, or renal arteries. CT of the kidneys with contrast (Figure 1) shows a slightly hypodense, roughly circular lesion in the lower pole of the right kidney, measuring approximately 1.8 * 1.8 cm, with a CT value of 19 HU. On contrast-enhanced scanning, the lesion has clear margins, exhibits heterogeneous hypodensity, and shows more prominent peripheral enhancement than central enhancement. Phase CT values: 38–55/41–82/54–90 HU. Renal dynamic scan + glomerular filtration rate (GFR) (Figure 2): 1. Slightly diminished contrast enhancement in both perfusion and functional phases of the right renal lower pole, consistent with CT findings suggestive of right lower pole pathology; no significant abnormalities in overall right renal function, with impaired upper right urinary tract drainage. 2. No significant abnormalities in left renal function. 3. Relative percentage of renal function: Left 54.31%, Right 45.69%. Renal Acoustic Contrast: 1. Solid mass lesion in the middle-upper pole of the right kidney (tumor—renal angiomyolipoma cannot be excluded); 2. Right main renal artery shows variable width (thinner than contralateral); Right adrenal artery appears thin throughout—suggestive of fibromuscular dysplasia; 3. No significant abnormalities in renal arterial blood flow. Renal vascular ultrasound findings: No significant abnormalities in renal artery flow velocity bilaterally, as shown in Table 2. Renin activity measurements from bilateral renal vein samples are presented in Table 3: Right:Left = 3.09:1.

**TABLE 2:** Bilateral renal artery Doppler ultrasound.

Renal artery	Vs (cm/s)	Vd(cm/s)	RI
Right renal trunk at renal portal	146.3	59.3	0.59
Right renal trunk mid-segment	119.7	58.2	0.51
Right renal artery origin	91.1	42.3	0.54
Right renal artery segment	90.3	45.8	0.49
Right renal artery interlobar branch	66.1	29.2	0.58
Right adrenal artery at renal portal	69.7	31.9	0.54
Right adrenal artery origin	103.4	48.6	0.53
Right adrenal artery segment	52.3	25.6	0.51
Right adrenal artery interlobar artery	59.7	33.5	0.44
Left renal artery at renal hilum	98.6	39.3	0.6
Left renal artery origin	92.7	38.6	0.58
Left renal artery segmental artery	64.4	27.5	0.57
Left renal artery interlobar artery	64.5	30	0.53

**TABLE 3:** Bilateral renal vein blood sampling results.

Position	Renin (pg/mL)	Angiotensin II (ng/mL/h)	Aldosterone (pg/mL)	Aldosterone/renin ratio (0–37)
Left	131.9	13.4	241.25	1.83
Right	408.07	41.47	255.69	0.63

Based on the patient’s clinical manifestations and relevant ancillary tests, a clinical diagnosis of “reninoma” was established. Further investigations revealed no absolute contraindications for surgery. Under ultrasound guidance, a right renal tumor biopsy was performed, and specimens were obtained. Subsequently, percutaneous radiofrequency ablation of the right renal tumor was conducted under ultrasound guidance, with the procedure completed successfully. Postoperative pathology report confirmed reninoma. Immunohistochemistry: CD117(+), CD34(+), CgA(−), CKpan(−), Desmin(−), Ki−67 (1% +), PAX−8(−), S−100 (diffuse +), SMA(−), Syn(−), Vimentin(+), β−catenin (partial +).

The diagnosis of reninoma requires a combination of clinical manifestations and pathological examination results. Most scholars believe that reninoma originates from glomerular arteriole vascular smooth muscle cells, with its main components including epithelioid cells, small vessels, and capillaries (1). Immunohistochemical staining of reninoma tissue demonstrates specific expression of renin by tumor cells, while vimentin and CD34 show diffuse positivity. GATA3, CD117, smooth muscle actin, and vimentin are expressed to varying degrees, and vascular endothelial growth factor is positive in some cases (2). Sirohi et al. pointed out that strong expression of SMA is favorable for the diagnosis of perivascular cell tumors, whereas strong positivity for CD34 and CD117 supports the diagnosis of paraglomerular cell tumors (3).

This tumor also requires differential diagnosis from multiple other neoplasms, including hemangioblastoma, hemangioid tumor, and epithelioid angiosarcoma. Hemangioblastoma shares overlapping immunophenotypes with reninoma, but GATA3 and renin are often positive in juxtaglomerular cell tumors but not expressed in hemangioblastoma. Hemangioid tumors are characterized by dilated thin-walled vessels and typically do not cause hypertension clinically, allowing for differentiation from juxtaglomerular cell tumors. Epithelioid angiosarcoma is immunohistochemically positive for HMB-45 and/or Melan-A in tumor cells, with varying degrees of positivity for smooth muscle markers, while Renin and CD34 are negative, facilitating differentiation (4, 5). The pathological immunohistochemistry in this case showed CD117(+), CD34(+), vimentin(+), and partial β-catenin(+), supporting the diagnosis of reninoma.

Follow-up: The patient’s blood pressure returned to normal postoperatively, with monitored readings of 100–110 mmHg systolic and 70–80 mmHg diastolic, requiring no medication. At the 6-month follow-up, the patient’s supine aldosterone and renin activity were normal: renin 51.73 pg/mL, aldosterone 120.76 pg/mL, angiotensin activity 2.26 ng/mL/h, aldosterone/renin ratio 2.33. Serum potassium levels were within normal limits, and renal ultrasound revealed no abnormalities. Two years postoperatively, the patient presented again with elevated blood pressure. Ambulatory blood pressure monitoring revealed a 24-h systolic peak of 178 mmHg and trough of 114 mmHg, diastolic peak of 116 mmHg and trough of 62 mmHg, with renin at 72.3 pg/mL and aldosterone at 262 pg/mL. Following comprehensive evaluation, tumor recurrence was suspected, and the patient underwent laparoscopic partial right nephrectomy. Postoperatively, the patient’s blood pressure normalized.

## 3 Literature search

Using the search terms “reninoma” and “juxtaglomerular cell tumor,” we searched the China National Knowledge Infrastructure (CNKI), Wanfang, and PubMed databases for relevant Chinese publications from 2016 to May 2025. A total of 18 Chinese and English articles were retrieved, comprising 62 cases with complete clinical data suitable for analysis. The clinical characteristics of the 62 patients summarized from the literature are presented in Table 4.

**TABLE 4:** Clinical data of 62 patients with reninoma.

Literature	Number of cases	Gender	Age	Duration of illness	BP (mmHg)	sK (mmol/L)	Rn activity [ng/(mL⋅h)]	AII (ng/L)	Aldo (ng/L)	Renal vein sampling	Tumor size (cm)	Surgical approach	Outcome
1 (15)	1	M	16	9m	170/100	3.6	–	S:129.75	NL	–	1.4 × 1.1	L-PN	3m post-op, NL Rn, BP. F/U CT: no Rcr.
2 (2)	1	F	16	1m	180/110	2.50↓	–	S:334. 63, L:135. 66	–	–	3 × 1.5 × 1.2	L-PN	1w post-op, NL BP, sK, Rn, AII, and Aldo.
3 (6)	1	M	14	2m	190/140	2.50↓	–	–	L:795.08	–	1.0 × 1.0 × 0.7	L-PN	3m post-op, NL BP, Rn, and sK. F/U CT: no Rcr.
4 (14)	1	F	20	5 y	220/130	2.95↓	S:27.46, L:26.50	–	S:948.5, L:398.4	NL	1.5 × 1.2	L-PN	1w, post-op, NL sK, Rn, and Aldo. BP was maintained at 120–148/79–108 mmHg with oral nifedipine controlled-release tablets.
5 (7)	9	M	35	–	180/112	3.5	6	262	403.97	–	2	PN	NL BP, sK, Rn, AII, and Aldo.F/U: 241m no Rcr.
		M	37	–	142/105	3.7	3.65	53.6	151.26	–	5.2	N	229m post-op, NL BP, sK, Rn, AII, and Aldo.
		M	22	–	190/140	3.5	12	134.4	306.86	–	1	PN	139m post-op, NL BP, sK, Rn, AII, and Aldo.
		M	22	–	165/125	3.09↓	12	159	205.78	–	0.9	Radiofrequency Ablation	61m post-op, NL BP, sK, Rn, AII, and Aldo.
		M	18	–	150/70	3.7	3.59	83.8	NL	–	1.9	PN	96m post-op, NL BP, sK, Rn, AII, and Aldo.
		M	12	–	155/95	2.80↓	9.41	800	364.62	–	2.8	PN	94m post-op, NL BP, sK, Rn, AII, and Aldo.
		F	28	–	171/115	3.5	12	155	158.84	–	6.7	N	54m post-op, NL BP, sK, Rn, AII, and Aldo.
		F	25	–	145/98	3.2↓	12	142	187.73	–	3	PN	53m post-op, NL BP, sK, Rn, AII, and Aldo.
		F	18	–	170/120	2.15↓	12	175	NL	–	1.8	PN	33m post-op, NL BP, sK, Rn, AII, and Aldo.
6 (16)	14	M	16	2y	220/140	3↓	>12	–	–	–	2.5	L-PN	13m post-op, NL BP, sK, Rn.
		M	46	6y	125/80	4.3	–	–	–	–	2.0	PN	NL BP, sK.
		F	37	7y	200/140	2.30↓	>12	–	–	–	4.8	L-PN	3m post-op, NL sK and Rn activity, BP 135/85 mmHg.
		F	28	1m	240/130	3.8	>12	–	–	–	4.2	PN	NL sK and Rn activity, BP 130/80 mmHg.
		F	40	12y	140/95	3.8	–	–	–	–	1.5	L-PN	84m post-op, NL sK and Rn activity, BP 132/94 mmHg.
		F	29	1m	180/145	2.50↓	NL	–	–	–	0.7	L-PN	84m post-op, NL sK and Rn activity, BP 130/95 mmHg.
		M	10	2y	209/180	2.80↓	>12	–	–	–	1.2	L-PN	NL BP and sK.
		F	34	4y	170/100	3.00↓	>12	–	–	–	1.1	L-PN	3m post-op, NL BP, sK and Rn activity.
		F	29	1y	220/180	2.80↓	>12	–	–	–	1.2	L-PN	6m post-op, NL sK and Rn activity, BP 130/80 mmHg.
		M	39	11y	180/120	2.70↓	18	–	–	–	1.1	L-PN	108m post-op, NL sK, Rn activity and BP.
		F	26	4m	200/110	3.9	NL	–	–	–	1.8	PN	NL sK and BP.
		F	15	1y	245/135	2.50↓	NL	–	–	–	1.0	PN	NL sK, Rn activity and BP.
		F	72	3y	130/80	3.8	–	–	–	–	2.9	PN	60m post-op, NL sK and BP.
		F	28	4y	200/120	3.10↓	NL	–	–	–	0.7	L-PN	3m post-op, NL sK, BP and Rn activity.
7 (17)	1	F	22	7d	210/130	3.20↓	–	L:156, S:147	S:1015↑, L:NL	–	2.1 × 2.0 × 1.9	L-PN	NL Rn and BP.
8 (18)	1	F	51	2y	145/99	3.99	–	–	–	–	2.5	L-PN	–
9 (19)	2	F	18	3y	160/110	3.32↓	11.5	–	–	–	3.4 × 3.5 × 2.8	PN	6m post-op, NL sK, BP and Rn.
		M	18	6m	220/130	3.00↓	4.99↑	150.33	NL		2.4 × 2.5 × 2.8	L-PN	15m post-op, NL sK, BP and Rn.
10 (20)	1	F	31	1y	200/120	3.30↓	>12	8	NL	–	1.7 × 2.2	L-PN	3m post-op, NL sK, AII, Aldo and BP and Rn.
11 (21)	1	F	36	7y	240/130	2.70↓	–	–	–	–	3.5 × 3.0 × 3.0	PN	1w post-op, NL sK and BP. F/U:18m no Rcr.
12 (10)	1	F	12	3m	150/106	3.01↓	17.48	211	697	Positive, right-sided dominance	7	L-PN	1w post-op, NL sK, BP, Rn, AII, and Aldo.
13 (22)	1	F	27	4y	160/96	2.80↓	NL	229.33	2988.29	–	1.4 × 1.0 × 0.5	PN	1w post-op, NL BP, Rn, AII, and Aldo.
14 (23)	1	F	17	10d	200/100	3.00↓	S:10.66, L:10.46	–	S:941.7, L:363.5	–	3.5 × 3.8 × 2.8	L-PN	–
15 (24)	1	F	28	20d	229/159	2.97↓	–	–	S:613, L:NL	–	3.5 × 3 × 2.5	L-PN	5m post-op, NL sK, BP, Rn, AII, and Aldo.
16 (8)	4	F	21	1m	150/110	2.86↓	–	–	593	–	2.0 × 1.5 × 1.5	L-PN	6m post-op, NL sK, BP, Rn and Aldo.
		F	53	7d	–	2.90↓	–	–	–	–	1.5	PN	–
		F	26	3y	230/120	3.00↓	–	–	–	–	3	PN	1m post-op, NL sK, BP, Rn, AII, and Aldo.
		F	28	–	–	NL	–	–	–	–	2.5	L-PN	F/U:11m no Rcr.
17 (25)	6	F	22	8y	150/100	2.93↓	L:17, S:20.5	–	NL	Positive, right-sided dominance	2.1 × 2.2 × 2.7	L-PN	42m post-op, NL sK, BP, Rn, AII, and Aldo.
		F	27	1y	130/90	3.48↓	L:9.1, S:12	–	S:NL, L:193	NL	2.7 × 2.3 × 2.0	L-PN	24m post-op, NL sK, BP, Rn, AII, and Aldo.
		F	17	16d	120/70	2.80↓	L:9.8, v12.8	–	S:NL, L:175	–	3.3 × 1.8 × 1.6	L-PN	22m post-op, NL sK, BP, Rn, AII, and Aldo.
		M	30	1m	170/100	2.70↓	L:7.8, S:12.5	–	S:NL, L:258	NL	1.8 × 1.6 × 1.6	L-PN	16m post-op, NL sK, BP, Rn, AII, and Aldo.
		M	28	7y	240/140	2.53↓	L:15.9, S:16.4	–	S:338, L:334	–	4.2 × 3.8 × 4.6	L-PN	8m post-op, NL sK, Rn, AII, and Aldo. BP still requires oral medication.
		M	23	4y	120/100	3.16↓	L:2.7, S:7.7	–	S:NL, L:185	–	3.2 × 2.6 × 2.8	L-PN	4m post-op, NL sK, BP, Rn, AII, and Aldo.
18 (26)	15	5M10F	Mean23 (10, 72)	Median 27 m (1 m to 12 y)	14 cases of hypertension were present.	10 patients had hypokalemia.	9 cases tested for Rn activity, all showed elevated levels.	–	11 cases tested Aldo levels, with 10 showing elevated levels.	–	1.5(0.9–5.9)	L-PN	NL BP in 13 patients. F/U: 6m to 15y, no Rcr in 15 patients.

F, Female; M, Male; d, days; w, weeks; m, months; y, years; NL, normal; S, standing; l, Lying; L-RN, Laparoscopic Radical Nephrectomy; PN, Partial nephrectomy; N, Nephrectomy; L-PN, Laparoscopic Partial Nephrectomy; Rn, renin; BP, blood pressure; post-op, postoperatively; F/U, follow up; CT, CT scan; AII, angiotensin II; Aldo, aldosterone; sK, serum potassium; Rcr, recurrence. ↑ for high, ↓ for low, – for not applicable.

## 4 Discussion

Reninoma, also known as juxtaglomerular cell tumor, is a rare endocrine tumor that secretes renin, leading to excessive activation of the renin-angiotensin-aldosterone system and resulting in significant secondary hyperaldosteronism. Aldosterone promotes sodium retention and potassium excretion, causing renal potassium loss and resulting in hypokalemia. This creates the clinical trial of “three highs and one low”: hypertension, elevated plasma renin activity, high aldosterone levels, and hypokalemia (2). A review of case data in the literature shows that reninoma primarily occurs in females, with a male-to-female ratio of approximately 1:2. The age of onset varies, with most cases occurring between 15 and 35 years old, and only six patients older than 40 years. To date, the youngest reported case in China was 9 years old, while the present case is 10 years old, making it extremely rare.

Hypertension is typically the earliest and most prominent clinical manifestation in patients, often presenting as resistant hypertension. Monotherapy often shows poor efficacy, and multiple antihypertensive drugs need to be used in combination to control blood pressure. Patients presenting for care may exhibit hypertension-related symptoms such as headache, dizziness, and blurred vision. Additionally, some patients may experience symptoms associated with hypokalemia, including fatigue. Clinically, reninoma is categorized into three types based on the patient’s blood pressure and serum potassium levels:—Typical reninoma: Patients with both hypertension and hypokalemia.—Atypical reninoma: Patients presenting with either hypertension or hypokalemia alone.—Non-functional reninoma: Patients lacking both hypertension and hypokalemia but pathologically confirmed to have a reninoma (6, 7). The patient in this case had hypertension for over a year, with multiple electrolyte tests showing no hypokalemia, indicating an atypical reninoma. This may relate to the tumor’s renin secretion activity and the body’s compensatory potassium metabolism mechanisms. During peak secretion, the tumor produces large amounts of active renin, activating the RAAS system, increasing aldosterone secretion, and enhancing renal potassium excretion. and elevated urinary potassium excretion rate. When the tumor is in a non-peak phase or its renin secretion activity decreases, the body activates regulatory functions to reduce potassium excretion, maintaining dynamic equilibrium. Among 62 patients reported in previous literature, 44 presented with typical reninoma, 16 with atypical reninoma (where patients exhibited hypertension but normal serum potassium levels), and 2 with non-functional reninoma. The latter two patients were admitted due to a right renal mass detected during physical examination. Following comprehensive investigations, they underwent laparoscopic partial nephrectomy, with postoperative pathology confirming reninoma (8).

Among 60 patients with hypertension, 43 exhibited varying degrees of elevated plasma renin activity, while 12 did not undergo complete renin activity testing (see Table 4). Therefore, elevated renin activity holds diagnostic significance for reninoma, but its diagnosis must be differentiated from other vascular diseases and renal masses that can cause increased plasma renin activity, including renal artery stenosis and renal cell carcinoma (9). Previous studies have suggested that in patients with a high clinical suspicion of reninoma, bilateral renal vein sampling for renin activity measurement may be performed. A bilateral renal vein renin activity ratio > 1.5:1 is diagnostic. Among the 62 patients in this study, 5 underwent bilateral renal vein sampling, with only 2 testing positive (40% positivity rate). Zhenqiu Yu et al. reviewed 110 Chinese patients diagnosed with reninoma before 2016, where 14 underwent bilateral renal vein sampling for renin activity measurement, yielding a positivity rate of only 21.4%. Studies indicate that unilateral renal vein sampling exhibits low sensitivity for diagnosing reninoma, potentially due to reninomas are mostly located in the renal cortex near the capsule, and their blood drainage may occur through the renal capsular veins rather than the main renal vein (5, 10). Additionally, when renal artery stenosis is present concurrently, the diagnostic value of measuring renin activity via unilateral renal vein sampling becomes limited. Differentiation requires combining bilateral renal artery flow velocity measurements with renal dynamic scanning.

The renal CTA findings in this case revealed partial narrowing of the bilateral renal arteries. MR angiography also demonstrated that the right bilateral renal arteries were narrower than the contralateral side. Complementary renal dynamic scanning and vascular ultrasound showed no abnormalities in right renal function or blood flow velocity in both renal arteries. This excludes hypertension caused by right renal artery stenosis activating the RAAS system. Therefore, the clinical diagnosis is a right reninoma.

Reninoma is predominantly a benign tumor, and surgical intervention remains the optimal treatment approach. Surgical options include nephrectomy, partial nephrectomy, and tumor resection. Postoperative local recurrence and metastasis are uncommon, with over 90% of patients no longer requiring antihypertensive medication after surgery (11). To maximally preserve renal function, nephron-sparing surgery is typically chosen when available (12). Among 62 patients over the past decade, 43 chose partial nephrectomy via laparoscopy. However, due to variations in tumor size and location, complex tumors—such as large tumors, completely endogenous renal tumor, or tumors near the renal hilum—require careful consideration of the surgeon’s skill level, the patient’s physical condition, and hospital facilities when choosing the surgical approach (13). Literature reports radiofrequency ablation as a viable treatment for small renal cell carcinomas extending into the cortex that are difficult to resect surgically. Among the summarized cases, only two patients were chosen for radiofrequency ablation. Among the two patients, one had an endophytic tumor at the renal hilum and underwent laparoscopic microwave ablation of the right renal tumor. At the 6-month postoperative follow-up, the tumor size was similar to preoperative measurements. Two years later, the patient presented again with headaches and elevated blood pressure, revealing marked tumor enlargement with hydronephrosis. Laparoscopic right nephrectomy was subsequently performed (4, 10). In this case, the clinical diagnosis was confirmed, and ultrasound-guided radiofrequency ablation of the right renal tumor was performed. Postoperative immunohistochemical pathology supported the diagnosis of a reninoma. Two years after the surgery, the patient presented again with elevated blood pressure. Partial nephrectomy ultimately normalized blood pressure, mirroring findings in the literature. This suggests that while radiofrequency ablation is minimally invasive, it has limitations in treating reninomas, potentially leaving residual tumor requiring surgical intervention. Among the 62 patients reviewed, most achieved normalized postoperative blood pressure, serum potassium, and renin levels. Eight patients required ongoing medication to control blood pressure. Literature reports indicate approximately 10% of patients still require medication postoperatively, potentially due to renal vascular lesions caused by long-term hypertension. Vigilance for tumor residuals or recurrence is also required (14).

For non-functional reninoma, patients often lack the significant features of hypertension and hypokalemia, requiring postoperative pathological confirmation (27). This may be related to the renin-secreting activity of the tumor. Although tumor cells originate from juxtaglomerular cells, their secretory function may be altered, with an excessively high proportion of inactive renin secreted, insufficient to cause clinical symptoms (28). The pathogenesis of atypical reninoma remains unclear, though current theories suggest a potential link to compensatory mechanisms in potassium regulation. Therefore, clinicians should evaluate for renin-mediated hypertension in any patient with early-onset, severe, or resistant hypertension and be aware of reninoma as a rare but very treatable cause. Postoperative outcomes are generally favorable with rare metastasis or recurrence. Regular postoperative follow-up is essential to monitor blood pressure, serum potassium levels, and renal imaging changes.

## Data Availability

The original contributions presented in the study are included in the article/supplementary material, further inquiries can be directed to the corresponding author.
